# Hospital burden of pulmonary arterial hypertension in France

**DOI:** 10.1371/journal.pone.0221211

**Published:** 2019-09-19

**Authors:** Emmanuel Bergot, Lucie De Leotoing, Hakim Bendjenana, Charlène Tournier, Alexandre Vainchtock, Gaëlle Nachbaur, Marc Humbert

**Affiliations:** 1 Service de Pneumologie & Oncologie Thoracique, Centre Hospitalier Universitaire de Caen, Caen, France; 2 Unicaen, UFR santé, Caen, France; 3 HEVA, Lyon, France; 4 GlaxoSmithKline, Rueil-Malmaison, France; 5 Université Paris-Sud, Faculté de Médecine, Université Paris-Saclay, Le Kremlin Bicêtre, France; 6 Assistance Publique Hôpitaux de Paris, Service de Pneumologie, Hôpital Bicêtre, Le Kremlin Bicêtre, France; 7 Inserm UMR_S 999, Le Kremlin Bicêtre, France; Azienda Ospedaliero Universitaria Careggi, ITALY

## Abstract

**Background & aims:**

Pulmonary arterial hypertension is a severe disease associated with frequent hospitalisations. This retrospective analysis of the French medical information PMSI-MSO database aimed to describe incident cases of patients with pulmonary arterial hypertension hospitalised in France in 2013 and to document associated hospitalisation costs from the national health insurance perspective.

**Methods:**

Cases of pulmonary arterial hypertension were identified using a diagnostic algorithm. All cases hospitalised in 2013 with no hospitalisation the previous two years were retained. All hospital stays during the year following the index hospitalisation were extracted, and classified as incident stays, monitoring stays or stays due to disease worsening. Costs were attributed from French national tariffs.

**Results:**

384 patients in France were hospitalised with incident pulmonary arterial hypertension in 2013. Over the following twelve months, patients made 1,271 stays related to pulmonary arterial hypertension (415 incident stays, 604 monitoring stays and 252 worsening stays). Mean age was 59.6 years and 241 (62.8%) patients were women. Liver disease and connective tissue diseases were documented in 62 patients (16.1%) each. Thirty-one patients (8.1%) died during hospitalisation and four (1.0%) received a lung/heart-lung transplantation. The total annual cost of these hospitalisations was € 3,640,382. € 2,985,936 was attributable to standard tariffs (82.0%), € 463,325 to additional ICU stays (12.7%) and € 191,118 to expensive drugs (5.2%). The mean cost/stay was € 2,864, ranging from € 1,282 for monitoring stays to € 7,285 for worsening stays.

**Conclusions:**

Although pulmonary arterial hypertension is rare, it carries a high economic burden.

## Introduction

Pulmonary arterial hypertension (PAH) is a rare, severe, chronic, potentially fatal disease characterised by pulmonary vascular resistance and elevated pulmonary arterial pressure which may lead to right ventricular failure [1–4]. Despite treatment advances, survival of patients with advanced PAH remains poor and recent data from the REVEAL Registry indicate that 5-year survival is somewhat higher than 60% [5]. The prevalence of PAH ranges between 15 and 50 per million and its incidence is around five cases per million per year [6–8].

Given the rarity of PAH, collecting data on the specific characteristics of patients with the condition, on risk factors and on the course of the disease is challenging, and patient registries have been set up in many countries to meet these challenges [6, 7], for example in France [8], Great Britain and Ireland [9], Spain [10], Germany with a contribution from other European centres (COMPERA) [11, 12] and the USA [13].

In France, the national reference centre for pulmonary hypertension established a network of seventeen university hospitals responsible for creating a national PAH Registry in the early 2000s [8]. The initial objectives of this Registry were to describe prospectively the frequency and clinical characteristics of PAH and to describe the evolution of PAH over a three-year follow-up period. All adult patients with a diagnosis of PAH consulting one of the participating centres over a twelve-month period in 2002–2003 were enrolled. Overall, 674 prevalent cases and 121 incident cases were identified and characterised.

Economic studies on the direct or indirect costs of PAH are scarce [14, 15]. In particular, the French Registry did not collect data on the economic burden of PAH, or provide information for this burden to be reconstituted retrospectively. Given that healthcare resources use and costs are highly dependent on the organisation and funding of the healthcare system, it is important to generate economic data at a national level. In this respect, analysis of data from medico-administrative databases is an attractive approach for collecting economic information on rare diseases such as PAH more rapidly and simply than could be done by setting up a dedicated Registry. Several such studies have been performed in the USA using insurance claims databases [14, 16–19]. The French National hospital database (*Programme de Médicalisation des Systèmes d’Information* database; PMSI) contains exhaustive medico-administrative information on care delivery for all hospital stays in France, coded by the medical condition for which the patient was hospitalised. This database has been widely used over recent years for determining the direct medical costs of different medical conditions [20].

The present study consists of an analysis of the PMSI database in order to identify all hospital stays related to new incident cases of PAH in France in 2013, to describe the patients and to document associated costs of hospitalisation.

## Materials and methods

### Study design

This was a retrospective analysis of a French medical information database (GSK study HO-15-16391). Data was extracted from the national Hospital medical information database (PMSI). The study population consisted of all patients hospitalised for PAH in France in 2013.

### PMSI database

The French PMSI-MCO (Medicine, Surgery, Obstetrics) database covers all overnight or day hospitalisations in the public and private sectors involving short-term stays in medical, surgical or obstetric facilities. Each patient in the database is attributed a unique anonymous patient identifier. This identifier can be used to track individual patients across multiple hospitalisations.

At the time of final discharge, a standardised discharge summary (SDS) is issued which lists all hospital procedures undergone by the patient during the stay, identified through standardised procedure codes. The reason for hospitalisation is identified by a diagnosis-related group (DRG) code, based on the International Classification of Diseases, 10th revision (ICD-10) [21], which is used by the hospital administration for costing purposes. Three different types of DRG code may be attributed to an individual stay. The principal diagnosis (PD) corresponds to the condition for which the patient was hospitalised (for example, myocardial infarction); the related diagnosis (RD) corresponds to any underlying condition which may have been related to the PD (for example, coronary artery disease); the significantly-associated diagnosis (SAD) corresponds to comorbidities or complications which may affect the course or cost of hospitalisation (for example, chronic kidney failure). All medical procedures are listed, including surgery, diagnostic tests and other examinations, and the departments in which the patient was hospitalised during the stay are documented. Most medications and non-pharmacological treatments cannot be specifically identified since they are integrated into the DRG cost. However, delivery of certain expensive drugs and recorded in a linked database (FICHCOMP) and can thus be identified individually. Socio-demographic information is limited to gender, age and postcode of residence. No information is available on the outcome of any procedure or the results of any test.

### Case selection

In a first step, all adult patients (≥ 18 years) hospitalised in France with pulmonary hypertension documented as a PD/RD/SAD between 1^st^ January and 31^st^ December 2013 were identified from the PMSI-MCO database. Pulmonary hypertension was documented by the presence of either ICD-10 code I27.0 *“Primary pulmonary hypertension”* or ICD-10 code I27.2 *“Other secondary pulmonary hypertension”* in the standardised discharge summary (SDS). The first hospitalisation in 2013 with one of these ICD-10 codes was considered as the index hospitalisation.

In order to capture incident cases only, all hospitalisations occurring in the two years prior to the index hospitalisation were examined retrospectively and cases with prior hospitalisations with pulmonary hypertension documented in the SDS were excluded. Since the ICD-10 codes are for pulmonary hypertension in general, and not only for PAH, it was necessary to identify cases with ‘presumptive’ PAH using a selection algorithm. This combined decision rules related to surrogate variables for a diagnosis of PAH established *a priori* by two clinical experts with a medical review of the SDS on a case-by-case basis by the same two experts.

In a first step, all hospital stays of at least one night during the year following the index hospitalisation were extracted from the database. Next, all stays (index hospitalisation and any subsequent stay) were screened for documentation of (1) right heart catheterisation, which is a recommended diagnostic and monitoring procedure for PAH [22], using the classification codes for medical procedures (S1 Table), (2) delivery of a prostacyclin analogue, which is only approved for the treatment of PAH or (3) lung/heart-lung transplantation. Patients fulfilling any of these three criteria were retained by the algorithm.

In the next step, the SDS were screened for any mention indicative of a pulmonary hypertension diagnosis other than PAH. These mentions could be either ICD-10 codes (such as D860 *“Sarcoidosis of lung”*, I260 *“Pulmonary embolism with mention of acute cor pulmonale”*, J961 *“Chronic respiratory failure”*), or procedure codes (such as left valvular surgery, pulmonary endarterectomy). These cases were excluded.

The remaining stages of the algorithm focussed on follow-up stays only, in order to retain only those related to PAH management. Examples of conditions or procedures which were considered to be related to PAH were M340 *“Progressive systemic sclerosis”* and R060 *“Dyspnoea”*. Stays for which such conditions or procedures were documented as the PD or RD were retained automatically by the algorithm. When they were documented as a SAD, stays were evaluated and adjudicated on a case-by-case basis by the clinical experts. All stays not considered to be related to PAH were excluded. For example, patients with secondary PH (for example due to left heart disease) were eliminated at this stage. In a final step, patients with no follow-up stays related to PAH management (as defined above) were excluded from the analysis.

### Classification of stays

All retained stays were then classified into three groups based on the context of hospitalisation, namely inclusion, monitoring, and worsening, according to the timing and duration of the stay and the reason for hospitalisation (Table 1). According to European practice guidelines [22, 23], all patients with PAH should be hospitalised for monitoring three and twelve months after diagnosis. Short hospital stays intervening in these periods were thus considered as stays for monitoring, unless the PD/RD code suggested otherwise. Where the assignment of the case was ambiguous, the stay was extracted from the database and assessed by a medical reviewer, who adjudicated the stay on the basis of information in the SDS. Stays were also classified on the basis of the objective of the hospitalisation into non-invasive medical management, surgery, minimally invasive procedures, and others (Table 1).

**Table 1:** Classification of stays.

***Context of hospitalisation***	
Inclusion	▪ First PAH stay documented in the database ▪Second PAH stay if on same or following day and length of stay < 5 days (unless death)
Monitoring	▪ Stay occurring 2–4 months or 11–12 months after the inclusion stay with a LOS < 3 days (unless Polymyositis (M332) as PD/RD for hospitalisation) ▪ Stay occurring 0–2 months or 4–11 months after the inclusion stay with a LOS < 3 days and exploration for PD/RD for hospitalisation (unless Polymyositis (M332) as PD/RD for hospitalisation) ▪ LOS < 5 days ▪ Exploration as reason for hospitalisation ▪ Right heart catheterization (unless congestive cardiac failure) ▪ Unless anaemia, cardiac arrest, dyspnoea, atrial flutter/fibrillation, respiratory failure) ▪ LOS > 1 day (unless cardiac/mitral failure) ▪ Occurrence < 11 days ▪ Occurrence > 10 days (unless interstitial pulmonary diseases, bradycardia, syncope and collapse, management of catheterization, dyspnoea, embolism, cardiac arrest, pleural effusion, respiratory failure, atrial flutter/fibrillation, cardiac failure, phlebitis ▪ Unless death
Worsening	▪ All stays with death of the patient (except index hospitalisation) ▪ All other stays
***Reason for hospitalisation***	
Non-invasive medical management	All non-invasive management (*eg* observation, pharmacological treatment, physiotherapy)
Surgery	All therapeutic surgical procedures (*eg* open-heart surgery, lung/heart-lung transplantation)
Minimally invasive procedures	Invasive procedures for diagnostic or monitoring purposes (*eg* catheterisation, endoscopy)
Others	*Eg* palliative care, chemotherapy

LOS: length of stay.

### Data collection

The socio-demographic characteristics of patients were collected (age at inclusion, gender). Comorbidities potentially associated with PAH were documented on the basis of ICD-10 codes associated with the hospital stays. A prespecified list of potential comorbidities of interest was established after medical review of DRG codes in the SRS was established, covering four disease groups, namely human immunodeficiency virus (HIV) infection, respiratory diseases, liver diseases, and connective tissue disorders.

For each hospital stay, the hospital sector (public or private), the type of hospital (university, community, clinic), the type of hospitalisation (day or overnight hospitalisation) and the length of stay were documented. Day hospitalisation was identified as a length of stay (LOS) of 0 days. In-hospital death, lung/heart-lung transplantation and prostacyclin therapy occurring during the study period were identified as outcome markers.

Expensive drug use (principally prostacyclins) was documented from the FICHCOMP database for each hospital stay of interest. It should be noted that, at the time of study completion, this database was only available for public hospitals. No extrapolation was performed from public hospitals to private hospitals, given the fact that 99.9% of prostacyclins prescribed in French hospitals in 2013 were administered in the public sector [24].

### Costing

Costing was restricted to direct costs and determined from the perspective of the French social security system (National Health Insurance; NHI). All costs are expressed in Euros. Costs were attributed from official French national tariffs for medical acts applicable in France in 2013 and 2014. These costs were updated to 2017 values to take account of inflation. A standard national tariff was applied to each hospitalization based on the DRG code attributed in the PMSI database. These standard tariffs include medical and related procedures, nursing care, treatments (except specific expensive drugs), food and accommodation, and investment costs for hospitalised patients. Additional costs per day of hospitalisation in an intensive care unit were added to the DRG tariffs when appropriate. For private hospitals, where physicians are reimbursed on a fee-for-service basis, physician fees were identified from the ENCC (*Echelle Nationale des Coûts à Méthodologie Commune*, the French observatory of real-world spending on healthcare) and added to the DRG tariffs. Expensive drugs were costed using the public retail price.

### Statistical analysis

Descriptive analyses were performed. Continuous data were presented as mean values with standard deviation (SD) or median values with range (quartile 1 and quartile 3) and categorical data as frequency counts and percentages.

Statistical Analysis System software, version 9.2 for Windows (SAS Institute Inc., Cary, NC, USA) was used for all analyses.

### Ethics and role of the funding source

The study was funded in full by GlaxoSmithKline. The writing and preparation of this manuscript was funded by GlaxoSmithKline. Initial data analyses were undertaken by CT, who is an employee of HEVA (Lyon, France) and received funding from GlaxoSmithKline. Writing support was provided by CT and LdL of HEVA (Lyon, France), as well as by Adam Doble of Foxymed (Paris, France) and was funded by GlaxoSmithKline. All authors had complete access to the data that supports this publication. The corresponding author had full access to all of the data and the final responsibility to submit for publication.

The study was conducted in accordance with relevant international and French regulatory requirements. Patient information in the PMSI database is fully anonymised before third parties are given access. Since this was a retrospective study of an anonymised database and had no influence on patient care, ethics committee approval was not required. Use of the PMSI-MCO database for this type of study has been approved by the French national data protection agency (CNIL; annual authorisation #1419102 v7–2015-111111-56-18 / order M14N056 and M14L056).

## Results

### Identification of cases of PAH and related hospitalisations

**Fig 1:**
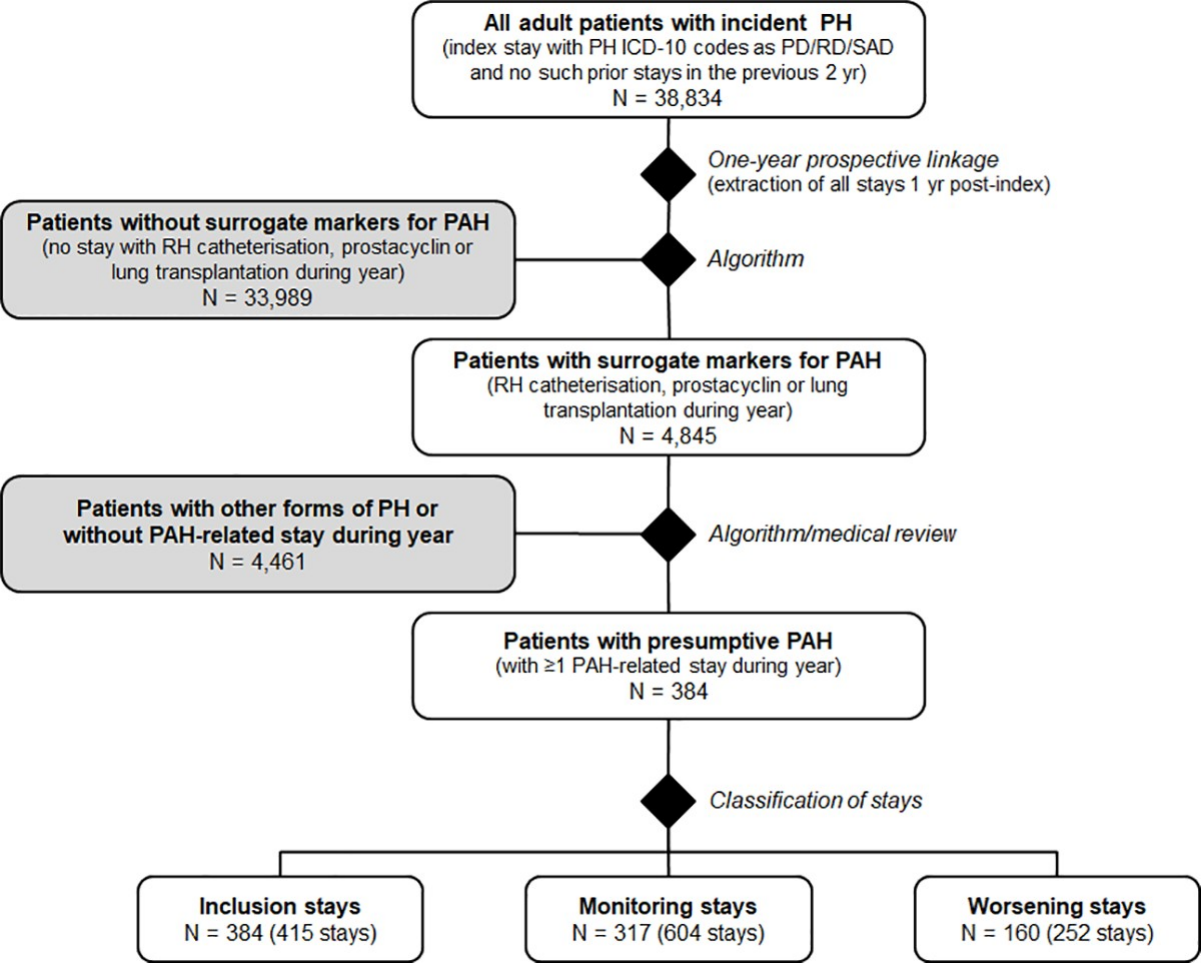
Flow chart of the study population.

In 2013, 38,834 incident adult patients with PAH ICD-10 codes were identified in the PMSI-MCO database (Fig 1). All hospital stays with PAH ICD-10 codes of these patients were extracted, over a period of one year after the first PAH stay in 2013 (the index hospitalisation). Right heart catheterisation, prostacyclin administration procedure and lung/heart-lung transplantation were not documented in any of the stays for 33,989 patients, and these were excluded from the cohort. The remaining 4,845 patients were reviewed by the Scientific Committee to exclude any concomitant pathology or procedure indicative of a diagnosis of another type of pulmonary hypertension. In the final step, all patients who did not have at least one follow-up stay for a pathology or procedure indicative of a diagnosis of PAH were excluded. The final cohort thus consisted of 384 patients with presumed PAH who made 1,271 stays for a reason related to PAH (full cohort).

By definition, all 384 patients had an inclusion stay. For some of them, the inclusion stay was immediately followed by another stay that did not fulfil criteria for a monitoring stay, and was considered an extension of the inclusion stay. Thus, the 384 patients had in total 415 inclusion stays. The remaining 856 stays were classified as 604 monitoring stays in 317 patients (82.6%) and 252 worsening stays in 160 patients (41.7%).

### Characteristics of patients with PAH

**Fig 2:**
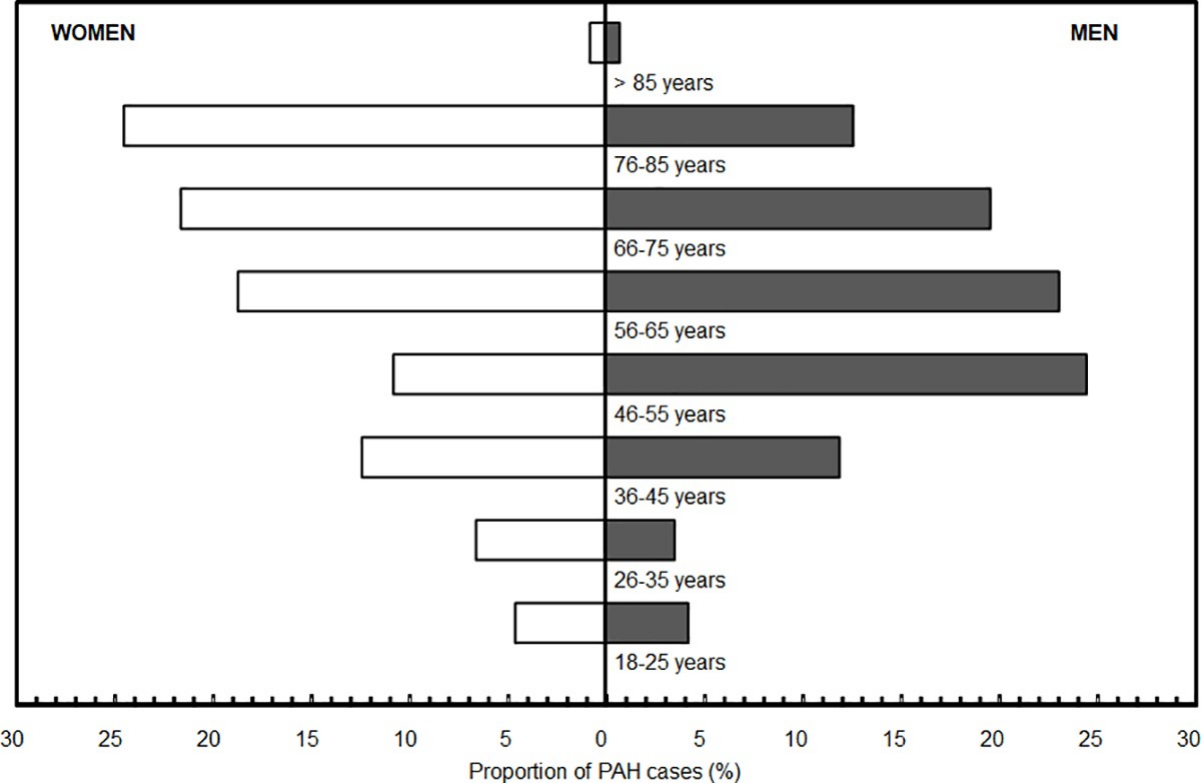
Distribution of PAH patients by gender and age (N = 384).

The characteristics of the 384 retained patients are presented in Table 2 and Fig 2. The mean age of the cohort at inclusion was 59.6 years and two-thirds were women. The most frequently documented comorbidities were liver diseases (16.1%) and connective tissue diseases (16.1%).

**Table 2:** Characteristics of 384 retained patients.

Age (years; mean ± SD)	59.6 ± 16.7
Gender (women: n, %)	241 (62.8%)
Comorbidities* HIV infection Respiratory diseases Liver diseases Connective tissue diseases	11 (2.9%) 18 (4.7%) 62 (16.1%) 62 (16.1%)

*Respiratory diseases included emphysema and chronic obstructive pulmonary disease. Liver diseases included alcoholic/toxic liver disease, hepatic failure, chronic hepatitis, fibrosis and cirrhosis of liver. Connective tissue diseases, included systemic lupus erythematosus, systemic sclerosis, other systemic involvement of connective tissue.

### Outcome markers

Of the 384 patients in 2013–2014, 31 died during hospitalisation, corresponding to an annual in-hospital case fatality rate of 8.1%. Four patients (1.0%) had a lung/heart-lung transplantation. Furthermore, forty patients (10.4%) were receiving prostacyclin during the study period. Only 8 patients of the cohort (2.1%) received prostacyclin at the inclusion visit, and 32 patients (8.3%) who were not taking prostacyclin at inclusion started it at a later follow-up visit.

### Characteristics of hospital stays

The characteristics of the 1,271 hospital stays made by the study cohort are presented in Table 3. Around half of the stays were classified as monitoring stays and one-fifth as stays associated with worsening of the clinical state of the patient (see Fig 1). Virtually all of the stays were in the public sector (96%) and three-quarters were in university hospitals. Day hospitalisation accounted for around a third of all hospitalizations and was most frequent for monitoring stays (N = 298; 49.3%) and very rare for worsening stays (N = 3; 1.2%). The mean length of stay associated with overnight hospitalizations was 6.7 days and was lowest for monitoring stays (2.1 ± 0.9 days) and highest for worsening stays (12.9 ± 12.9), consistent with the length of stay criterion for classifying the time of stay. Most of the stays were for non-invasive medical management (50.8%), followed by minimally invasive procedures (46.8%) and surgical procedures (0.8%). Other types of management accounted for only 1.6% of stays, and corresponded to one stay for palliative care (classified as a worsening stay) and 19 for chemotherapy for non-tumoral disease. The stays for surgery included the four cases of lung/heart-lung transplantation (all worsening stays).

**Table 3:** Characteristics of 1,271 hospital stays.

Hospital sector Public Private	1,224 (96.3%) 47 (3.7%)
Type of hospital University hospital Community general hospital Other public hospitals Private hospitals with public health missions Private not-for profit hospitals Private for-profit hospitals	981 (77.2%) 186 (14.6%) 10 (0.8%) 47 (3.7%) 4 (0.3%) 43 (3.4%)
Length of hospitalisation Day hospitalisation Overnight hospitalisation Length of stay (overnight hospitalisation only)	404 (31.8%) 867 (68.2%) 6.7 ± 9.0 days
Purpose of hospitalisation Non-invasive medical management Surgery Minimally invasive procedures Others	646 (50.8%) 10 (0.8%) 595 (46.8%) 20 (1.6%)

### Cost of hospitalisation for PAH

From the NHI perspective, the estimated total annual cost of hospital stays for PAH in French hospitals during 2013–2014 was € 3,640,382, of which € 2,985,936 was attributable to standard DRG tariffs (82.0%), € 463,325 to additional daily costs for ICU stays (12.7%), and € 191,118 to expensive drugs (5.2%). Prostacyclin administration represented 83.6% of the cost of all expensive drugs (€ 159,789).

The total annual cost was also estimated according to the context of stay. This cost was € 1,030,101 for the 415 inclusion stays (28.3% of total cost), € 774,452 for the 604 monitoring stays (21.3%), and € 1,835,829 for the 252 worsening stays (50.4%). The four lung/heart-lung transplantations cost € 360,737, representing 19.6% of the total worsening stays cost.

The mean cost per stay was € 2,864. This cost ranged from € 1,282 for monitoring stays to € 7,285 for worsening stays (Table 4). It was split into the following components: tariffs (82.0%), daily supplements such as ICU (12.7%), and expensive drugs (5.2%). Expensive drugs covered 9.5% of the mean cost per worsening stay, while they accounted for only around 1% of the mean cost per inclusion or monitoring stay.

**Table 4:** Mean and median cost (NHI Perspective) of PAH management in 2013–2014.

Stays		Cost per stay (NHI perspective)		Detail of mean cost per stay (%)		
		Mean ± SD	Median [range]	Standard DRG tariff	ICU	Expensive drugs
All stays	N = 1,271	€ 2,864 ± 5,981	€ 1,550 [826–3,269]	82.0%	12.7%	5.2%
Inclusion	N = 415	€ 2,482 ± 2,517	€ 1,550 [1,281–3,382]	87.5%	11.7%	0.8%
Monitoring	N = 604	€ 1,282 ± 723	€ 1,281 [787–1,550]	98.4%	0.4%	1.2%
Worsening	N = 252	€ 7,285 ± 11,974	€ 4,161 [3,269–6,510]	72.0%	18.5%	9.5%

SD: standard deviation; ICU: daily supplements

The mean cost was also analysed according to the type of management (Table 5). Surgical procedures were associated with the highest mean cost per stay overall, and in all contexts except monitoring where surgery was not used. The only “other management” in the ‘worsening’ group was a palliative care stay ending with death of the patient (length of stay 114 day) and costing € 26,429. The seven surgical procedures in the ‘worsening’ group were four lung/heart-lung transplantations (€ 357,277), one thoracoscopy respiratory intervention (€ 21,965), one case of cardiac assistance (€ 8,567), and one pacemaker implantation (€ 6,666).

**Table 5:** Mean cost (NHI Perspective) of PAH management in 2013–2014 according to the type of management.

Stays	Mean (± SD) cost				
	Total	Non-invasive medical management	Surgery	Minimally invasive procedures	Others
All stays	€ 2,864 ± 5,981 (N = 1,271)	€ 2,527 ± 3,411 (N = 646)	€ 41,321 ± 43,078 (N = 10)	€ 2,623 ± 3,080 (N = 595)	€ 1,694 ± 5,882 (N = 20)
Inclusion	€ 2,482 ± 2,517 (N = 415)	€ 2,057 ± 1,677 (N = 189)	€ 4,973 ± 959 (N = 3)	€ 2,854 ± 3,022 (N = 219)	€ 366 ± 0 (N = 4)
Monitoring	€ 1,282 ± 723 (N = 604)	€ 980 ± 619 (N = 291)	- (N = 0)	€ 1,622 ± 664 (N = 298)	€ 382 ± 14 (N = 15)
Worsening	€ 7,285 ± 11,974 (N = 252)	€ 5,774 ± 5,151 (N = 166)	€ 56,899 ± 42,890 (N = 7)	€ 5,800 ± 5,601 (N = 78)	€ 26,685 *(no SD)* (N = 1)

SD: standard deviation.

## Discussion

This study aimed to identify all patients hospitalised for PAH in France in 2013 and to document the costs of these hospitalisations. Overall, 384 patients with a first hospitalisation for PAH were identified. The identification of these patients in the PMSI database is challenging, firstly because the condition is rare, and secondly because the characteristic diagnostic hallmarks of PAH, mean pulmonary arterial pressure and pulmonary vascular resistance, are not documented in the database. For this reason, the approach was firstly to identify all cases of pulmonary hypertension using the relevant ICD-10 codes in the SDS and secondly to identify the cases of ‘presumptive’ PAH by the surrogate marker of hospitalisations for right heart catheterisation, which is a specific diagnostic procedure for PAH. Patients with ICD-10 codes indicative of other forms of pulmonary hypertension were also excluded. This tiered approach was necessary since hospitalisations for pulmonary hypertension in general are relatively frequent, as a complication of many common diseases [7]. This strategy has been followed successfully in previous studies of insurance claims databases in the USA [25, 26]. Other North American studies have used prescription of specific PAH medications as a surrogate measure for identifying cases in claims databases [14, 17, 19]. In studies of the PMSI database, such as the present one, such an approach is limited to expensive medications documented in the FICHCOMP database, such as prostacyclin.

The use of an algorithm and surrogate markers for case identification without the possibility of clinical ascertainment through the medical records will necessarily be not entirely accurate. Possible sources of misattribution of cases in our study include patients for whom right heart catheterisation is performed and found to be inconsistent with a diagnosis of PAH, or for whom this procedure is performed for other reasons than diagnosis of PAH; such patients would be incorrectly assigned as cases. However, the SDS of nearly all the hospital stays of all patients retained by the algorithm were reviewed on a case-by-case basis by two chest physicians in order to identify false positives, as far as the information allowed, and to exclude them. In contrast, patients who were not monitored by right heart catheterisation, as recommended in practice guidelines, would be missed, as would patients who die at home before returning for a follow-up visit and patients who choose not go to hospital for the recommended follow-up visits for monitoring. In particular, it should be noted that although the European guidelines recommend monitoring with right heart catheterisation every six to twelve months (or more frequently in case of treatment changes or clinical worsening), in everyday practice patients with mild disease severity who are adequately controlled on their current treatment may not undergo this invasive procedure so frequently on a routine basis, and so will not be hospitalised nor captured by the algorithm. It is not known what proportion of patients with PAH this would represent.

Our study identified 384 incident cases of PAH. This is higher than the number of incident cases reported ten years previously in the French Registry (121 patients over a one-year recruitment period) [8], although the investigators of the latter indicated that their finding was likely to be an underestimate, as only seventeen hospitals participated in the Registry, and 64% of the cases came from the French National Reference Centre for Pulmonary Hypertension. The promoters of this Registry emphasised that patients with PAH might not be referred to pulmonary vascular centres participating in the Registry, particularly if they were being followed for other serious diseases or if they were being treated in the community with oral drugs. In principle, all patients with PAH should have been captured in the present study, whether they were hospitalised in a specialised pulmonary vascular centre or elsewhere. However, in terms of age, gender and presence of connective tissue diseases, our patients were very similar to those reported in the French Registry [8]. It was not possible to compare haemodynamic data or outcome of functional tests between the two samples since these are not documented in the PMSI database.

Since information on clinical severity is unavailable in the PMSI database, New York Hospital Association (NHYA) functional class cannot be assigned. NYHA Functional Class I patients are excluded *de facto* from the cohort because these patients are infrequent and not usually hospitalised. For this reason, such patients would thus not appear in the PMSI database with a hospitalisation related to PAH. The study population therefore consisted of NYHA Functional Class II, III and IV patients. Many patients with PAH who fall into NYHA Functional Class IV or advanced NYHA Functional Class III can be identified by the surrogate marker of prostacyclin administration, since this drug is usually prescribed for such advanced cases. This is the only PAH therapy that can be identified, as it is reimbursed separately and identified in the FICHCOMP database. This was the case for 10.4% of patients (N = 40) in the study cohort. However, this is likely to be an underestimate due to underuse of prostacyclin in some severe cases.

The study provided the first estimate available for France on the costs of hospitalisations associated with PAH. The estimated total annual cost of these hospitalisations was € 3,640,382, corresponding to a mean cost per stay of € 2,864. Half of this cost was expended on stays associated with clinical worsening. Lung/heart-lung transplantations were performed in four patients (1.0%) at a cost of € 360,737, which represented 9.9% of the total annual cost. Even though prostacyclins were only prescribed to 10.4% of patients, they accounted for 4.4% of total cost. These findings indicate that improvements in management of patients with PAH that would reduce the probability of hospitalisation for disease worsening or obviate or reduce the need for lung/heart-lung transplantation could reduce the economic burden of PAH.

These hospitalisation costs can be compared to those of other common cardiovascular or pulmonary diseases in France that have been derived from medico-administrative databases over the last decade using a similar methodology. The mean cost per patient is lower than that of other chronic cardiovascular or pulmonary disorders, such as COPD (€ 6000) [27] or acute heart failure (€ 4713) [28], principally because most of the stays are monitoring stays. Hospital stays for worsening of PAH, on the other hand, are more expensive than hospitalisations for exacerbations of chronic obstructive pulmonary disorder (COPD) or acute heart failure (AHF). However, given the much lower prevalence of PAH than these disorders, the total cost of hospitalisations to the health system (€ 4 million) is much lower than in the case of COPD exacerbations (€ 670 million) or AHF (750 million). The total cost of hospitalisation for PAH thus represents around 0.02% of the total cost of all hospitalisations in France (~ € 73 billion in 2015) [29]. Of this total hospitalisation cost, cardiovascular diseases account for ~€ 8 billion and pulmonary diseases ~€ 1 billion [29].

Our cost findings are difficult to compare with those of the North American costing studies due to differences in the organisation and funding of the health system, and in the scope of costs considered. However, they can be compared with recent costing studies from Belgium [30] and Germany [31]. In an analysis of a hospital medico-administrative database in Belgium conducted in 2013 [30], cost of unscheduled (*ie* not for monitoring) hospital stays of 35 patients with PAH were evaluated and provided a mean cost per stay of €20,229, which is three times higher than the costs estimated for worsening stays in France over the same period. The mean length of stay was also longer in the Belgian database (17 days versus 13 days). The German study [31] was a microcosting study which evaluated both hospital and community costs accrued by a sample of 142 patients with PAH between 2004 and 2006. In this study, the mean cost per hospital stay was €1,523 (inpatient stays) and €267 (outpatient visits), which are somewhat lower than in our more recent French study, even taking into account cost inflation over the intervening decade.

This study has several strengths and limitations. The principal strength is the exhaustiveness of the source database, covering all hospitalisations in the public and private sectors in France, which, in principle, means that all cases of this rare disease are captured. In addition, information of all in-hospital health resource consumption is exhaustive, except for non-costly drugs (as they are not reported inside the DRGs), and documented in a standardised fashion, from which costs from the payer perspective can be determined using national standard tariffs. The principal limitation is the lack of clinical ascertainment of case assignment, as discussed above, potentially leading to misallocation of cases. In addition, costs can only be determined for in-hospital resource consumption, whereas community costs, such as primary care consultations, monitoring in community clinics and prescription of medication for use at home are not documented. The German cost evaluation has indicated that these community costs, notably for drug prescription, contribute significantly to the economic burden of PAH.

In conclusion, analysis of the PMSI database provides complementary information to patient registries for the identification of all hospitalisations associated with PAH, and to estimate the ensuing costs. The findings confirm that although PAH is a rare condition, it carries a high economic burden.

## Supporting information

S1 Table CCAM codes for right heart catheterization. (DOCX)
