# Supplementary material for: Hospital burden of pulmonary arterial hypertension in France
Source: PLoS One. 2019 Sep 19;14(9):e0221211. doi: 10.1371/journal.pone.0221211 (PMC6752797; doi:10.1371/journal.pone.0221211)
Supplement: S1 Table — (DOCX) [file pone.0221211.s001.docx]

**Supporting Information**

S1 Table. CCAM codes for right heart catheterization

| CCAM code | French wording |
| --- | --- |
| EQQF006 | *Mesure et enregistrement des pressions du cœur droit et de l'artère pulmonaire, sans injection de produit de contraste, par voie veineuse transcutanée* |
| EQQH001 | *Mesure et enregistrement des pressions du cœur droit et de l'artère pulmonaire, avec injection de produit de contraste, par voie veineuse transcutanée* |
| EQQF001 | *Mesure et enregistrement des pressions du cœur droit, de l'artère pulmonaire et du cœur gauche, sans injection de produit de contraste, par voie veineuse transcutanée et par voie artérielle transcutanée ou cathétérisme du foramen ovale* |
| EQQH005 | *Mesure et enregistrement des pressions du cœur droit, de l'artère pulmonaire et du cœur gauche, avec injection de produit de contraste, par voie veineuse transcutanée et par voie artérielle transcutanée ou cathétérisme du foramen ovale, avant l'âge de 24 mois* |
| EQQH006 | *Mesure et enregistrement des pressions du cœur droit, de l'artère pulmonaire et du cœur gauche, avec injection de produit de contraste, par voie veineuse transcutanée et par voie artérielle transcutanée ou cathétérisme du foramen ovale, à l'âge de 24 mois ou plus* |
| EQQF004 | *Mesure et enregistrement des pressions du cœur droit, de l'artère pulmonaire et du cœur gauche, sans injection de produit de contraste, par voie veineuse transcutanée avec perforation du septum interatrial* |
| EQQH004 | *Mesure et enregistrement des pressions du cœur droit, de l'artère pulmonaire et du cœur gauche, avec injection de produit de contraste, par voie veineuse transcutanée avec perforation du septum interatrial* |
| EQQF003 | *Mesure et enregistrement des pressions intravasculaires pulmonaires et systémiques et des différences artérioveineuses des contenus artériels en oxygène, à l'état basal et après administration d'agent pharmacologique vasodilatateur, par voie vasculaire transcutanée* |
| EQLF004 | *Pose de dispositif de mesure des pressions du cœur droit et du débit cardiaque, par voie veineuse transcutanée* |

(CCAM – Classification Commune des Actes Médicaux; French classification for procedures).
